# What evidence exists for the environmental outcomes of nature-based aquaculture systems? a systematic map protocol

**DOI:** 10.1186/s13750-026-00387-8

**Published:** 2026-06-28

**Authors:** Thirukanthan Chandra Segaran, Mohamad Nor Azra, Mohd Iqbal Mohd Noor, Nor Afiqah-Aleng, Li Lian Wong, Wen Jye Mok, Mazlan Abd Ghaffar, Tengku Sifzizul Tengku Muhammad, Min Pau Tan, Muhd Danish Daniel Abdullah, Teoh Shwu Jiau, Aileen Tan Shau Hwai, Edward Hugh Allison, Yeong Yik Sung

**Affiliations:** 1 Higher Institution Centre of Excellence (HICoE), Institute of Climate Adaptation and Marine Biotechnology (ICAMB), Universiti Malaysia Terengganu, Kuala Nerus, 21030 Terengganu Malaysia; 2 Faculty of Business and Management, Universiti Teknologi MARA (UiTM), Puncak Alam Campus, 42300, Selangor, Malaysia; 3 WorldFish, Jalan Batu Maung, Bayan Lepas, 11960 Penang Malaysia; 4 Centre for Marine and Coastal Studies, Universiti Sains Malaysia, Penang, 11700 Malaysia

**Keywords:** Ecosystem-based aquaculture, Oyster reefs, Integrated multi-trophic aquaculture, Macroalgae cultivation, Nature-inspired solutions, Nature-positive aquaculture

## Abstract

**Background:**

Nature-based solutions (NbS) are defined as actions that protect, conserve, restore, and sustainably use natural or modified ecosystems. These actions aim to deliver environmental services, enhance biodiversity, and support societal well-being. NbS are increasingly promoted across a wide range of sectors, including agriculture, coastal management, and civil engineering. However, despite being introduced almost twenty years ago, NBS appear not to have been widely and explicitly adopted in aquaculture production systems policies and practices, arguably because there is little evidence of their efficacy in improving environmental outcomes and production. This systematic map therefore seeks to address the primary review question: What evidence exists in the literature on reported environmental outcomes associated with nature-based approaches in aquaculture? To answer this question, the study will identify, collate, and characterize the available evidence on nature-based approaches applied within aquaculture systems.

**Methods:**

This systematic map will follow a structured evidence-synthesis approach to identify and characterize literature on nature-based solutions in aquaculture. Comprehensive searches will be conducted across established scientific databases using carefully developed search strings, and these will be complemented by targeted grey literature searches across specialist aquaculture and NbS platforms, as well as Google Scholar. Retrieved records will be screened at title, abstract, and full-text levels against predefined eligibility criteria. Studies will be included if they focus on aquaculture systems, examine interventions framed as or consistent with NbS, report outcomes related to environmental performance, socio-economic value, or policy relevance, and are based on empirical evidence from peer-reviewed or credible grey literature sources. Data from eligible studies will be extracted and summarized descriptively, followed by a narrative synthesis to identify thematic clusters, geographical patterns, and gaps in the evidence base. The outputs will provide a structured knowledge base to inform researchers, practitioners, and policymakers seeking to expand the application of NbS in aquaculture.

**Supplementary Information:**

The online version contains supplementary material available at https://doi.org/10.1186/s13750-026-00387-8.

## Background

The Anthropocene era presents humanity with interconnected global challenges, notably climate change mitigation and adaptation, biodiversity conservation, and ensuring human well-being. Addressing these issues separately can lead to unintended negative outcomes due to their intrinsic interconnectedness [40]. Hence, integrated approaches, aligned with the ethos of the United Nations Sustainable Development Goals (SDGs), emphasizing connectivity, inclusivity, and synergistic partnerships, are crucial [56].

Currently, environmental crises pose unprecedented threats to both nature and human society, manifested through intensified climate change, global warming, extreme heat waves, air and water pollution, rising greenhouse gas emissions, and zoonotic pandemics, all contributing significantly to global morbidity and mortality [14, 45]. These stressors disrupt ecological stability, resulting in habitat destruction, accelerated biodiversity loss, compromised access to clean air and water, reduced food security, and declining human quality of life [43]. Therefore, urgent action towards sustainable strategies, effective conservation practices, and eco-friendly transformations is imperative [49].

According to the World Economic Forum’s Global Risks Report [9], extreme weather events and natural disasters constitute the greatest threats to global economic stability and human health, both in terms of their severity and frequency. Crucially, the report highlights inadequate climate mitigation and adaptation efforts as factors exacerbating these threats [16, 46]. Understanding nature’s potential role in addressing these vulnerabilities requires adopting a comprehensive vulnerability framework for social-ecological systems, as established by the Intergovernmental Panel on Climate Change (IPCC). This framework integrates ecological and socioeconomic vulnerabilities through three dimensions: (i) exposure to climate impacts, (ii) sensitivity of systems to these impacts, and (iii) adaptive capacity or resilience in responding to changing conditions [33, 46, 53].

In this context, nature-based solutions (NbS), defined as actions leveraging natural ecosystems and processes to address societal challenges, have emerged as an integrative strategy capable of minimizing trade-offs and promoting synergy across SDGs [23, 46]. Unlike conventional ‘hard engineering’ or ‘silver bullet’ technology-based interventions, NbS may offer cost-effective opportunities to simultaneously address climate adaptation and mitigation while delivering multiple ancillary benefits for society and ecosystems [58].

There is growing evidence across multiple sectors, including land management, coastal protection, wastewater treatment, urban planning, biodiversity conservation, and water management, that nature-based solutions (NbS) can deliver multiple benefits for both people and ecosystems [17, 27, 36, 38, 42, 60]. NbS have also been shown to contribute effectively to climate change adaptation strategies [7] and disaster risk reduction [58]. These “living solutions” integrate natural processes and structures to address complex societal and environmental challenges simultaneously, generating substantial ecological, social, and economic co-benefits [18].

This growing evidence for the efficacy of NbS has increased their prominence within international climate policy frameworks. Notably, the Paris Agreement, under the United Nations Framework Convention on Climate Change (UNFCCC), explicitly recognizes ecosystems as critical for climate change mitigation and adaptation. It emphasizes the preservation and enhancement of greenhouse gas sinks and reservoirs, and ecosystem integrity, particularly highlighting oceans, forests, and biodiversity protection [47, 48].

Today, NbS increasingly extend beyond ecological stewardship and are perceived also as pathways for sustainable economic diversification and transformation [15]. Despite this growing momentum, their application and outcomes in aquaculture remain underexplored. This paper aims to critically examine the application of the NbS concept in aquaculture, with particular focus on evaluating the environmental outcomes of aquaculture that advances, or claims to advance, NbS. The paper first briefly outlines the definition of NbS and its evolution as an influential concept shaping contemporary sustainable development policy discourses. Next, the paper introduces the application of NbS concepts in aquaculture and highlights its archetypes-integrated multi-trophic aquaculture and seaweed, shellfish-reef and mangrove-based systems –outlining the claims for the potentially positive outcomes, for both people and nature, of adopting and expanding these forms of aquaculture. We then move beyond the claims and counterclaims of advocates and skeptics to develop a systematic mapping protocol of the literature on NbS in aquaculture. The specific aim is to characterize and organize the existing evidence base, with particular attention to how environmental outcomes have been studied, reported, and measured across different nature-based approaches in aquaculture.

### Definitions of nature-based solutions

The term “nature-based solutions” (NbS) first appeared in the literature through a 2008 World Bank publication [31]. Although this foundational document introduced the term internationally, it lacked a formal, explicit definition. Instead, NbS was implicitly defined through the report’s practical applications, framing various World Bank initiatives that leveraged ecosystem management, such as forests, wetlands, and coral reefs, as practical tools to achieve climate adaptation and sustainable development objectives [31, 50]. The inferred definition from this early conceptualization described NbS as:

“Actions and investments where the conservation and sustainable management of ecosystems are used as functional, pragmatic tools to help societies, particularly in developing countries, adapt to the impacts of climate change and achieve broader development objectives.”

Following its introduction by the World Bank, the term “nature-based solutions” was embraced by the International Union for Conservation of Nature (IUCN), transitioning from development finance to the heart of global conservation discourse. By 2009, the IUCN actively promoted NbS at international climate negotiations, notably during the UNFCCC COP15 in Copenhagen, where it reframed existing ecosystem-based methods as direct solutions to global climate issues, enhancing their policy appeal. This advocacy continued at the IUCN World Conservation Congress in 2016, where the first formal definition was established under Resolution WCC-2016-Res-069, reported by Cohen-Shacham et al. [10]. The official IUCN definition is:

“Actions to protect, sustainably manage, and restore natural or modified ecosystems, that address societal challenges effectively and adaptively, simultaneously providing human well-being and biodiversity benefits.”

Analyzing the IUCN definition reveals several key components. The terms “protect, sustainably manage, and restore” ground NbS firmly within traditional conservation activities. Acknowledging “natural or modified ecosystems” reflects realism concerning extensive human impact on global landscapes and recognition of the fallacy of ‘wilderness myths’ [1, 24]. Furthermore, the broad framing of “societal challenges” positions NbS beyond climate concerns alone, encompassing critical issues such as food security, disaster risk reduction, water management, and public health, thus aligning with the broad targets of the Sustainable Development Goals (SDGs). Importantly, by recognizing existing concepts such as ecosystem-based adaptation (EbA), ecosystem-based disaster risk reduction, and green infrastructure, the IUCN positioned NbS as a comprehensive “umbrella concept” (Nature-based Solutions Standard [37]).

Parallel to the IUCN’s definition, the European Commission introduced its interpretation of NbS through the Horizon 2020 research and innovation program in 2015. The European Commission definition emphasized the role of NbS in fostering economic growth, job creation, technological innovation, and resilience, extending its relevance significantly to urban contexts (IUCN, n.d. [26]).

The 2015 European Commission definition is [25]:

“Solutions that are inspired and supported by nature, which are cost-effective, simultaneously provide environmental, social and economic benefits and help build resilience. Such solutions bring more, and more diverse, nature and natural features and processes into cities, landscapes, and seascapes, through locally adapted, resource-efficient and systemic interventions.”

According to the European Commission, NbS are nature-inspired, cost-effective approaches providing simultaneous environmental, social, and economic benefits. These solutions enhance resilience and introduce diverse natural processes into urban, rural, and marine environments through locally tailored, resource-efficient interventions. Despite broad acceptance, the original European Commission definition faced scrutiny for potentially enabling activities detrimental to biodiversity, such as monoculture plantations for carbon sequestration [3].

Addressing these concerns, the European Commission refined its definition in 2020, mandating that NbS must yield net biodiversity benefits and support diverse ecosystem services [39].

The added clause was:

“Nature-based solutions must benefit biodiversity and support the delivery of a range of ecosystem services.”

The period from 2020 to 2022 marked the maturation of the NbS concept, moving from competing definitions toward global consensus and practical application. The IUCN developed a technical standard for practitioners, while high-level political dialogues under the United Nations Environment Assembly (UNEA) are formalizing the conceptual framework into global environmental policy.

The culmination of over a decade of conceptual evolution occurred on March 2, 2022, at the fifth session of the UN Environment Assembly (UNEA-5). Member states adopted the first and only multilaterally agreed-upon definition of NBS, cementing its place in international environmental law and policy. The definition was adopted in Resolution 5/5, titled “Nature-based solutions for supporting sustainable development” (UNEA [57]).

The globally agreed-upon definition reads:

“Actions to protect, conserve, restore, sustainably use and manage natural or modified terrestrial, freshwater, coastal and marine ecosystems which address social, economic and environmental challenges effectively and adaptively, while simultaneously providing human well-being, ecosystem services, resilience and biodiversity benefits.”

**Fig. 1:**
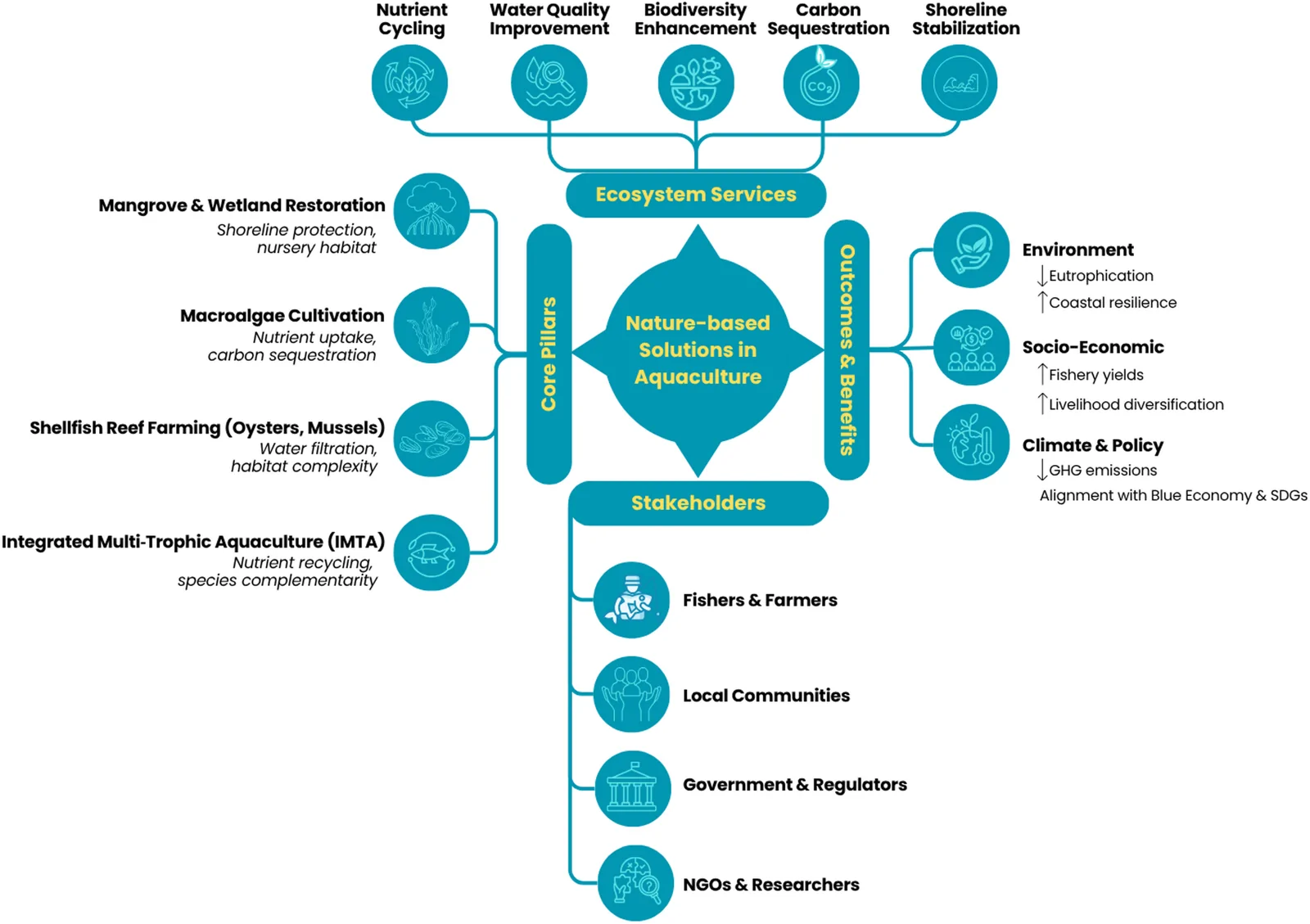
Conceptual framework of nature‑based solutions (NbS) in aquaculture. Four core NbS strategies (i) mangrove & wetland restoration (shoreline protection; nursery habitat), (ii) macroalgae cultivation (nutrient uptake; carbon sequestration), (iii) shellfish reef farming (oysters, mussels; water filtration; habitat complexity), and (iv) integrated multi‑trophic aquaculture (IMTA; nutrient recycling; species complementarity), drive five principal ecosystem services: nutrient cycling, water quality improvement, biodiversity enhancement, carbon sequestration, and shoreline stabilization. These services translate into outcomes across three domains: environmental, socio‑economic, and climate & policy. Effective co‑design and implementation of adaptive management are enabled by stakeholder engagement spanning fishers & farmers, local communities, government & regulators, and NGOs & researchers

### NbS in aquaculture

Nature-based solutions in aquaculture leverage natural ecosystems and processes to deliver multiple ecosystem services, significantly benefiting ecological sustainability, socio-economic prosperity, and climate resilience [29]. In this review, Fig. 1 is used to situate NbS approaches within a broader social-ecological context and to clarify how different intervention archetypes are linked to reported ecosystem services and outcome domains, without pre-empting the results of the systematic map. The figure does not imply that these approaches are the only forms of NbS in aquaculture, nor does it evaluate their relative effectiveness. Instead, it provides a consistent conceptual reference to support transparent mapping of how NbS are framed, studied, and reported across diverse aquaculture contexts.

A foundational ecosystem service definition by Bennett et al. [5] underscores these as benefits derived by humans from ecosystems, inherently emphasizing their role within social-ecological systems. Recognizing these interactions and interdependencies is crucial, as modifications to one ecosystem service can positively or negatively affect others. In aquaculture, the core pillars underpinning NbS include mangrove and wetland restoration, macroalgae cultivation, shellfish reef farming, and integrated multi-trophic aquaculture (IMTA).

One essential NbS approach in aquaculture is mangrove-integrated aquaculture, which is claimed to significantly enhance shoreline protection, mitigate climate change through blue carbon sequestration, and support fisheries productivity, biodiversity conservation, water quality, and cultural heritage [30, 55]. Historical evidence from the Chakaria Sundarbans in Bangladesh illustrates the severe ecological and socio-economic impacts of mangrove degradation due to shrimp farming expansion, underscoring the urgent need to reintegrate mangroves into aquaculture landscapes [2, 32]. Successful mangrove-integrated initiatives, as documented by Perera et al. [41], were implemented in Demak, Java, Indonesia, under the *Building with Nature* project and associated Green Mussel Culture initiatives. These interventions demonstrated substantial positive outcomes, including enhanced food security, livelihood improvement, coastal protection, and supplementary economic benefits. For example, near-shore fish populations showed marked recovery, and wild catch yields increased, contributing to improved community nutrition. Farmers adopting low external input sustainable aquaculture practices in shrimp ponds reported more than tripled gross margins, while additional income was generated through bio-rights and sustainable mussel farming established adjacent to regenerated mangrove zones. Successful mangrove-integrated initiatives, as documented by Perera et al. [41], were implemented in Demak, Java, Indonesia, under the *Building with Nature* project and associated Green Mussel Culture initiatives. These interventions demonstrated substantial positive outcomes, including enhanced food security, livelihood improvement, coastal protection, and supplementary economic benefits. For example, near-shore fish populations showed marked recovery, and wild catch yields increased, contributing to improved community nutrition. Farmers adopting low external input sustainable aquaculture (LEISA) practices in shrimp ponds reported more than tripled gross margins, while additional income was generated through bio-rights and sustainable mussel farming established adjacent to regenerated mangrove zones.

Building upon the ecosystem services highlighted by mangrove restoration, macroalgae cultivation has emerged as another potentially crucial NbS strategy within aquaculture. Historically integral to coastal livelihoods, seaweed utilization has diversified significantly beyond traditional food and feed purposes to encompass applications such as biomaterials, fertilizers, bioplastics, biofuels, and environmental detoxification [11, 13]. Contemporary studies emphasise macroalgae’s capacity to advance multiple Sustainable Development Goals (SDGs), providing ecosystem services such as nutrient absorption, carbon sequestration, and habitat enhancement, thereby contributing broadly to climate resilience, biodiversity, and socio-economic prosperity [20, 52]. However, the excitement over seaweed as a nature based climate mitigation solution must be tempered by critical analysis [4, 9]. There are, for example, on-going debates about the extent to which red seaweed as a ruminant feed additive can reduce methane emissions, with a recent experimental study on grazing beef cattle suggesting a 37% reduction is possible [35] and others suggestions that. Similarly, while seaweed cultivation has been proposed as a potential pathway for large-scale carbon sequestration through biomass sinking in the deep ocean, the feasibility and effectiveness of such approaches remain uncertain. Significant logistical, economic, and ecological challenges persist, and current understanding of marine carbon cycling processes is insufficient to confidently assess the long-term sequestration potential of this strategy [12, 28]. Global seaweed production is largely driven by aquaculture practices in Asia [6, 61]. This dominance highlights opportunities to expand macroalgae cultivation in other regions, particularly through the development of temperate and cold-water species farming systems [51]. However, significant uncertainties remain regarding the ecological limits of large-scale expansion, as well as the technological, societal, and economic challenges that may constrain the global growth of the sector [48].

In continuity with macroalgae cultivation, shellfish reef farming, especially involving oysters and mussels, represents a further robust application of NbS. These reefs function as natural coastal stabilizers, significantly mitigating erosion, enhancing coastal resilience, and reducing the frequency and associated ecological costs of sand replenishment activities [34, 59]. Shellfish perform critical ecosystem functions, including nutrient filtration and eutrophication control, thereby preventing harmful environmental outcomes such as fish mortality and toxic algal blooms [19, 34]. Additionally, they provide substantial cultural, recreational, and educational ecosystem services, further amplifying their socio-economic and ecological value [54].

Extending the principles demonstrated by the previously discussed NbS pillars, Integrated Multi-Trophic Aquaculture (IMTA) represents an advanced, ecosystem-based aquaculture practice. IMTA strategically integrates multiple trophic levels, imitating natural ecological interactions to maximize nutrient recycling and ecological efficiency [41]. China’s successful implementation of IMTA, constituting approximately 40% of its mariculture, underscores the viability and scalability of this approach, supported by adaptive governmental policies and nationwide integration [8, 22]. By incorporating diverse trophic species, such as finfish, mollusks, crustaceans, echinoderms, and aquatic plants, IMTA effectively mitigates nutrient pollution, optimizes resource use, and strengthens the resilience of aquaculture systems to climate-induced environmental stressors [44]. However, to date, evaluation of the environmental performance of IMTA, though case-studies analysed using newly developed Aquaculture Performance Indicators [21] have failed to show that it performs better, environmentally, than efficient intensive monoculture systems.

While together, these interconnected NbS approaches in aquaculture form a cohesive framework seemingly capable of addressing complex environmental challenges, socio-economic demands, and climate policy objectives, their uptake at scale is hindered by vested interests, transition costs and skepticism over claims of their efficacy.

### Stakeholders and expert engagement

This systematic map protocol was developed in response to a 2024 call for projects co-funded by Global Affairs Canada and the International Development Research Centre (IDRC). The work is led by the International Center for Living Aquatic Resources Management Inc. and WorldFish Headquarters (Malaysia) under the project entitled Climate-adaptive, inclusive, nature-based aquaculture (CAINA) in Malaysia and Solomon Islands, which forms part of the broader Nature-based Climate Solutions in Aquacultural Food Systems in Asia-Pacific (AQUADAPT) initiative.

Stakeholder and expert engagement was integrated throughout the development of the protocol to strengthen conceptual clarity, methodological rigor, and search sensitivity. A core group of academic experts was assembled from Universiti Malaysia Terengganu, Universiti Sains Malaysia, Universiti Kebangsaan Malaysia, Universiti Putra Malaysia, Universiti Malaysia Sabah, Universiti Malaya, Solomon Islands National University, and the Kastom Gaden Association. These experts were selected based on demonstrated experience in nature-based solutions, aquaculture systems, environmental assessment, and evidence synthesis, reflected through peer-reviewed publications, leadership of applied research projects, or institutional roles in NbS-related initiatives.

These experts were consulted at multiple stages of protocol development. Their input contributed to defining the scope of the systematic map, refining the conceptual framework, identifying dominant NbS archetypes in aquaculture, and developing inclusion and exclusion criteria. Expert feedback was also used iteratively to refine and validate the search strings, inform database selection, and structure outcome categories. In addition, selected experts contributed to the screening and coding stages to support consistency and transparency in study classification.

Beyond academic engagement, consultation extends to practitioner and institutional stakeholders during the evidence identification phase. Organizations such as the International Union for Conservation of Nature (IUCN), WorldFish, and national aquaculture research institutions will be contacted to identify unpublished reports, technical documents, and project outputs not readily accessible through academic databases. This combined engagement approach is intended to enhance geographic and thematic coverage, reduce publication bias, and ensure that the systematic map captures both academic and practice-oriented evidence relevant to nature-based aquaculture.

### Objective of the review

This systematic map protocol sets out the approach that will be used to collect and organize studies examining the environmental outcomes of aquaculture systems that apply nature-based practices. The review will identify and categorize interventions such as mangrove restoration, seaweed farming, shellfish reef cultivation, and integrated multi-trophic aquaculture. It will document how these interventions have been studied in relation to outcomes associated with water quality, biodiversity, nutrient cycling, carbon capture, and coastal protection, including the indicators and metrics used across the literature. The purpose is to map the scope and characteristics of existing evidence rather than to assess the effectiveness of individual interventions. It will also record the methods and indicators used to measure these outcomes. The overall goal is to provide an overview of the current evidence base, highlight geographical and thematic knowledge gaps, and support future research and planning for sustainable aquaculture systems.

### Research questions

These questions are designed to support a descriptive systematic map by characterizing the scope, distribution, and nature of the existing evidence base.

### Primary research question

What evidence exists in the literature on reported environmental outcomes associated with nature-based approaches in aquaculture?

### Secondary research questions

1. What types of nature-based approaches have been described or implemented in aquaculture systems across different aquatic settings?
2. What environmental outcomes are reported in relation to these approaches, and how are they described and measured?
3. In which geographical regions and ecological contexts have nature-based aquaculture approaches been applied?
4. What species, production systems, or system components are involved in these reported applications?
5. What tools, indicators, and methodological approaches are used to monitor and report environmental change in nature-based aquaculture systems?

## Method

This systematic mapping will follow the Collaboration for Environmental Evidence (CEE) Guidelines and Standards for Evidence Synthesis in Environmental Management and will adhere to the ROSES (Reporting Standards for Systematic Evidence Syntheses) reporting framework (see Additional File 1). The methodology is detailed in this protocol.

### Searching for articles

To identify relevant literature, this systematic map protocol will apply a comprehensive and structured search strategy covering peer-reviewed articles, books, theses, and grey literature. Searches will be conducted across selected academic databases, complemented by targeted searches of organizational and sector-specific websites. Citation tracking, including backward and forward searches, will be used to capture additional relevant studies not retrieved through database searches. Where full-text access is unavailable, authors will be contacted directly. Review articles identified during the search will be screened to locate relevant primary studies. The academic search platforms Web of Science Core Collection and Scopus will be systematically searched.

To further enhance search coverage, citation chasing will be employed. Forward and backward citation tracking will be used to identify additional relevant studies that may not have been captured in the main database searches. Tools like *citationchaser* (available at https://citationchaser.org/) will be used where appropriate to streamline this process. Additionally, a targeted grey literature search will be undertaken using Google Scholar, supplemented by searches on specialist platforms and relevant organizational websites.

### Search terms and languages

The search strategy is designed to prioritise sensitivity over precision, with the aim of capturing the broadest possible range of literature relevant to aquaculture systems and nature-based approaches. A wide set of aquaculture-related terms is used to encompass diverse production systems, cultured taxa, and farming practices across marine, brackish, and freshwater settings. These terms are combined with an inclusive set of keywords describing nature-based, ecosystem-based, and nature-inspired concepts, recognising that NbS are often not labelled consistently in the aquaculture literature.

The following search string will be applied in the *Title-Abstract-Keyword* (Scopus) and *Topic* (Web of Science) fields:

=(“aquacultur*” OR “aqua-cultur*” OR “aqua* cultur*” OR “freshwater cultur*” OR “marine cultur*” OR “sea cultur*” OR “ocean cultur*” OR “brackishwater cultur**” OR “brackish-water cultur*” OR “pen cultur*” OR “pond cultur*” OR “cage cultur*” OR “net pen* cultur” OR “aqua* farm*” OR “sea farm*” OR “marine farm*” OR “ocean farm*” OR “freshwater farm*” OR “brackishwater farm*” OR “brackish-water farm*” OR “aqua* pond*” OR “aqua* produc*” OR “sea cage* cultur*” OR “freshwater cage* cultur*” OR “marine cage* cultur*” OR “ocean cage* cultur*” OR “earth* pond cultur*” OR “earth pond farm*” OR “fish farm*” OR “shell* farm*” OR “crustace* farm” OR “mollus* farm” OR “mussel farm*” OR “oyster farm*” OR “shrimp farm*” OR “prawn farm*” OR “crayfish farm*” OR “lobster farm*” OR “crab farm*” OR “seaweed farm*” OR “fish pond*” OR “seaweed farm*” OR “macroalga* farm*” OR “macro-alga* farm*” OR “microalga* farm*” OR “micro-alga* farm*” OR “fish cultur*” OR “shell* culture*” OR “crustace* cultur*” OR “mollus* cultur*” OR “mussel cultur*” OR “oyster cultur*” OR “shrimp cultur*” OR “prawn cultur*” OR “crayfish cultur*” OR “lobster cultur*” OR “crab cultur*” OR “seaweed cultur*” OR “clam cultur*” OR “scallop cultur*” OR “abalone cultur*” OR “salmon cultur*” OR “tilapia cultur*” OR “seabass cultur*” OR “seabream cultur*” OR “trout cultur*” OR “catfish* cultur*” OR “carp cultur*” OR “clam farm*” OR “scallop farm*” OR “abalone farm*” OR “salmon farm*” OR “tilapia farm*” OR “seabass farm*” OR “seabream farm*” OR “trout farm*” OR “catfish* farm*” OR “carp farm*” OR “macroalga* culture*” OR “macro-alga* culture*” OR “microalga* cultur*” OR “micro-alga* culture*” OR “intensive culture” OR “extensive culture” OR “semi-intensive culture” OR “hatcher* culture” OR “tank culture” OR “net-pen culture” OR “grow out culture” OR “broodstock culture” OR “larviculture” OR “larv* culture” OR “nursery culture” OR “intensive farm*” OR “extensive farm*” OR “semi-intensive farm*” OR “hatcher* farm*” OR “tank farm*” OR “net-pen farm*” OR “grow out farm*” OR “broodstock farm*” OR “larv* farm*” OR “nursery farm*” OR “mariculture” OR “mari-culture” OR “pisciculture” OR “live food culture” OR “live food farm*” OR “Artemia culture” OR “rotifer* culture” OR “Brachionus culture” OR “copepod* culture” OR “Daphnia culture” OR “freshwater husbandry” OR “marine husbandry*” OR “sea husbandry” OR “ocean husbandry” OR “brackishwater husbandry” OR “brackish-water husbandry” OR “aquarium* culture” OR “aquarium* husbandry” OR “alga* cultur*”).

### AND

= (“natur* base* solution*” OR “natur*-base* solution*” OR “ecosystem* base* solution*” OR “ecosystem*-base* solution* OR “ecosystem* base* adaptation* OR “ecosystem*-base* adaptation*” OR “natur* depend* system” OR “natur* base*” OR “nature based strateg*” OR “natural climate solution*” OR “ecosystem friendly engineering” OR “building with nature” OR “working with nature” OR “nature derived solution*” OR “nature based feature*” OR “nature inspired solution*” OR “nature inclusive design*” OR “nature inspired design*” OR “nature derived design*” OR “ecosystem* based measure*” OR “ecosystem* based mitigation” OR “natural barrier*”).

The finalized search will be conducted on the Web of Science Core Collection and Scopus platforms, accessed through institutional subscriptions provided by Universiti Malaysia Terengganu. Only articles published in English will be included in the systematic map. This decision reflects practical constraints related to screening, data extraction, and interpretation, and is consistent with common practice in systematic maps where resources for translation are limited. The development of the search string is described in Additional File 2.

### Publication databases to be searched

The academic search platforms Web of Science Core Collection and Scopus will be systematically searched. Within Web of Science, searches will be conducted across the databases available through the authors’ institutional subscription, including the Science Citation Index Expanded and the Social Sciences Citation Index. For both Web of Science and Scopus, searches will cover publications from 2005 onwards. These platforms were selected based on preliminary scoping, which indicated strong coverage of relevant environmental and aquaculture literature.

The comprehensiveness of the final search string was evaluated using a predefined benchmark set comprising 10 key publications identified during the scoping phase. These benchmark studies represent well-cited and conceptually relevant examples of nature-based approaches in aquaculture and cover a range of intervention types, aquatic settings, and reported environmental outcomes.

### Estimating the comprehensiveness of the search

A total of ten benchmark articles were selected during the initial scoping phase based on expert knowledge of the field and their relevance to widely cited nature-based aquaculture interventions and associated environmental outcomes. These studies were chosen to reflect diversity in intervention types, aquatic settings, and outcome measures. The benchmark set was then used iteratively to refine the search strings by checking whether all benchmark articles were retrieved by the draft searches. Search terms were adjusted until the final search strategy successfully captured all benchmark studies, thereby providing confidence in the comprehensiveness of the search (see Additional File 2).

### Specialist searches

To capture non-indexed reports and grey literature, several organizational databases and websites will be systematically searched. These include sources maintained by governmental agencies, non-governmental organizations, and research institutions involved in implementing, monitoring, or funding nature-based solutions in aquaculture and coastal systems. Organizational databases typically consist of formal repositories, while organizational websites may provide less formal collections such as reports, case studies, technical guidelines, or policy briefs not indexed in academic search engines.

The following websites will be searched systematically:

- https://iucn.org
- https://www.naturebasedsolutionsinitiative.org
- https://www.nature-basedsolutions.com
- https://www.unepfi.org
- https://research-and-innovation.ec.europa.eu
- https://www.cabi.org
- https://www.un.org/en
- https://www.undrr.org
- https://www.unep.org
- https://www.ipbes.net
- https://worldfishcenter.org
- https://www.worldwildlife.org
- https://www.fao.org/home/en
- https://www.thegef.org
- https://www.copernicus.eu/en
- https://www.wri.org
- https://www.genevaenvironmentnetwork.org
- http://nature.org/en-us
- https://www.greenpolicyplatform.org
- https://aquaculturemag.com/
- https://thefishsite.com

### Article screening and study eligibility criteria

#### Screening process

The screening process will consist of two main phases: (1) title and abstract screening and (2) full-text screening. Both phases will be conducted independently by two reviewers. In the first phase, titles and abstracts will be screened for relevance based on predefined inclusion and exclusion criteria. Articles that meet the criteria will be retained for full-text screening. A pilot screening will be conducted prior to the main screening to assess consistency between reviewers. Thirty articles will be randomly selected and independently screened by two reviewers. A consistency check using Cohen’s Kappa statistic will be performed, with a minimum agreement threshold set at 75%.

Following the pilot, the remaining titles and abstracts will be screened independently and in parallel by the same two reviewers. When disagreements occur, articles will be marked as “Maybe” and referred to a third independent reviewer for final decision. In the second phase, full texts of all retained articles will be retrieved and screened to confirm final inclusion. Abstracts of the full texts will be reviewed collaboratively by all reviewers to determine eligibility for data extraction. Any discrepancies will be flagged and resolved through group discussion among all six reviewers to reach a consensus.

Full-text screening and data extraction will be conducted using a structured Google Form. Articles selected for inclusion will be distributed among the six reviewers for data extraction. Articles excluded at the full-text screening stage will be recorded together with the reasons for exclusion, which will be provided in Additional File 2. The structured Google Form used for full-text screening and data extraction will be provided as Additional File 3. All extracted data will be cross-checked by one author (MA) to ensure accuracy and consistency.

#### Eligibility criteria

To be included at both the title and abstract screening stage and the full-text screening stage, articles will be required to meet the eligibility criteria described below. These criteria will be defined according to the population, intervention, outcome, and study type elements of the systematic map.

#### Eligible population

This systematic map includes studies that focus on aquaculture systems located in marine, brackish, or freshwater environments. Eligible systems must involve the cultivation of aquatic organisms such as finfish, crustaceans, mollusks, or aquatic plants. These systems may operate in a variety of contexts, including cages, ponds, raceways, nearshore zones, estuaries, lagoons, and open water. Both intensive and extensive systems are eligible, provided they incorporate or interact with ecological components. Aquaria, laboratory-only systems, or closed recirculating systems without any environmental interface are excluded unless nature-based features are explicitly integrated.

Studies involving terrestrial, subterranean, or deep-sea ecosystems are outside the scope and will not be included unless they clearly describe an aquaculture setting that incorporates nature-based elements. If a study includes multiple types of aquaculture systems, it will be included if at least one of them fits the eligible criteria.

#### Eligible interventions

Eligible interventions must involve the application of nature-based practices or ecosystem-based methods within aquaculture systems. These may include, but are not limited to:

- Mangrove or wetland restoration associated with aquaculture.
- Seaweed or macroalgae cultivation used for nutrient uptake or habitat provisioning.
- Shellfish reef farming for water filtration or habitat enhancement.
- Integrated multi-trophic aquaculture systems.
- Use of biological components for sediment stabilization or shoreline protection.

To qualify, interventions must be purposefully implemented or managed to deliver environmental benefits, such as enhancing water quality or ecosystem functioning. Practices based solely on engineered, chemical, or mechanical solutions with no ecological foundation will be excluded. Hybrid systems that integrate ecological elements with infrastructure are eligible if the nature-based component plays a significant role in environmental performance.

#### Eligible comparators

This systematic map does not require a formal comparator for study inclusion. Eligible studies may report environmental outcomes with or without explicit comparison groups. When present, comparators may include spatial contrasts (e.g., sites with and without interventions), temporal contrasts (e.g., before and after implementation), or system-level comparisons (e.g., monoculture versus integrated systems). Comparative findings, when available, will be recorded to illustrate how NbS outcomes have been assessed relative to other practices, but the map will not independently evaluate whether NbS interventions perform “better” than conventional systems.

The classification of practices as NbS follows established archetypes in the literature, such as mangrove and wetland restoration, macroalgae cultivation, shellfish reef restoration, and integrated multi-trophic aquaculture. This categorization reflects their alignment with NbS principles of working with ecological processes to deliver multiple co-benefits, rather than an assessment of the technological form (e.g., longline vs. reef structures). The purpose of the mapping exercise is therefore to document and synthesize the evidence base for NbS in aquaculture, including where comparative studies exist, while acknowledging that judgments of relative performance are dependent on the design and outcomes of the individual studies.

#### Eligible outcomes

Studies must report at least one environmental outcome associated with the nature-based aquaculture intervention. Outcomes may include, but are not limited to:

- Water quality indicators (e.g. nutrient removal, turbidity reduction).
- Biodiversity or species composition changes.
- Habitat structure or complexity.
- Carbon sequestration or greenhouse gas mitigation.
- Sediment stability or coastal resilience.
- Functional ecological indicators (nutrient cycling, primary productivity).

Outcomes may be reported through quantitative data, observational assessment, or model-based estimation. Studies focusing solely on productivity, economics, or social factors without linking to environmental variables will be excluded unless environmental metrics are also reported.

#### Eligible types of study design

Empirical studies using observational, experimental, modeling, or quasi-experimental approaches will be included. These may be:

- Field studies measuring environmental conditions.
- Laboratory studies linked to environmental applications.
- Modeling studies calibrated with real-world data.
- Case studies with a described methodology.

Studies must be based on original data. Theoretical frameworks, conceptual discussions, editorials, commentaries, and narrative reviews will be excluded. Only peer-reviewed journal articles published in English will be included to ensure reliability and comparability.

### Study validity assessment

The objective of this systematic map is to provide a broad and structured overview of the existing evidence base on the environmental outcomes of nature-based aquaculture systems, with particular attention to the types of interventions, geographic coverage, and outcome metrics reported. As such, a formal assessment of study validity will not be conducted beyond the eligibility criteria described above.

However, to enhance the transparency and interpretability of the evidence base, we will extract and code specific attributes related to study design and methodological approach. These include the type of study (e.g. experimental, observational, modeling), the methods used to assess environmental outcomes, the frequency or duration of performance monitoring, and whether any form of comparator is reported. While not intended as a quality rating, these descriptors will support users in making preliminary assessments regarding the strength and applicability of the evidence. Limitations due to the absence of formal critical appraisal will be acknowledged in the final synthesis.

### Data coding strategy

Data coding in this systematic map will be guided by the aim to structure and classify the existing evidence on nature-based aquaculture systems in relation to their environmental outcomes. A standardized data extraction form will be developed collaboratively by the review team to ensure consistency across reviewers. This form will be piloted on a small sample of studies to refine the coding structure and address any ambiguities before full-scale data extraction begins.

For each included study, bibliographic details such as the title, authors, year of publication, journal, and country or region of the study will be recorded. The broader study context will also be captured, including geographic setting (such as tropical or temperate regions), the type of aquatic environment (marine, coastal, brackish, or freshwater), and the target ecosystem or habitat (e.g., mangroves, lagoons, estuaries). Aquaculture system characteristics will be coded based on the type of species farmed (finfish, shellfish, crustaceans, macroalgae), production scale (small-scale or commercial), and whether the system is monoculture or multi-species.

Nature based interventions will be coded according to the intervention type (such as mangrove restoration, seaweed cultivation, shellfish reef farming, or integrated multi trophic aquaculture), the purpose of the intervention (e.g., nutrient removal, biodiversity enhancement), and the design approach (passive or active intervention). Where available, details on the duration or timeline of the intervention will also be noted.

Environmental outcomes will be categorized based on the nature and scope of the reported results, including biodiversity changes, improvements in water quality, carbon sequestration, sediment stability, and other ecosystem functions. Each outcome will be linked to the type of measurement or indicator used, such as species richness, nutrient concentrations, or sedimentation rates. Where available, the direction and magnitude of the reported effect will be recorded.

In terms of study design and methods, each article will be coded for its research approach, including whether it is observational, experimental, modeling-based, or quasi-experimental. The presence or absence of a comparator (such as a control site or before-and-after comparison) will be noted, along with the data collection methods used (e.g., field sampling, laboratory analysis, remote sensing). The frequency and duration of environmental monitoring will be recorded to provide insight into the robustness and scope of performance assessments. Additionally, methodological clarity, including whether protocols are clearly described, and the type of data presented (quantitative, qualitative, or mixed methods), will also be documented.

Additional information such as references to ecosystem services, integration with local or national policy frameworks, and stated limitations or recommendations for future research will also be coded. All data will be entered into a shared online form and cross-checked by a second reviewer to ensure accuracy. Any discrepancies will be discussed and resolved collaboratively by the review team. This approach will support structured synthesis and allow for the generation of maps and visual summaries highlighting the distribution and characteristics of evidence across themes, geographies, and intervention types. All extracted data will be recorded using a shared online form (e.g. Google Form or spreadsheet platform). Entries will be cross-checked by a second reviewer to ensure consistency and reduce errors. Any discrepancies will be discussed among the review team and resolved through consensus. This approach will enable thematic analysis, evidence mapping, and visualization of trends, densities, and gaps within the global literature on nature-based aquaculture systems.

### Study mapping and presentation

Metadata from all included studies will be standardized and compiled into a structured dataset suitable for analysis. Analyses will be conducted to explore patterns in the distribution, characteristics, and focus areas of the evidence on nature-based aquaculture systems and their environmental outcomes. These analyses will be guided by the primary and secondary research questions. The evidence base will first be mapped by intervention type, such as mangrove restoration, seaweed farming, shellfish reef cultivation, and integrated multi-trophic aquaculture. For each intervention, we will document the reported environmental goals, the number of goals mentioned, and whether environmental outcomes are presented as the primary focus or as co-benefits. Co-benefits will be categorized into ecological, social, or economic domains.

Environmental outcomes will be grouped into thematic categories, including ecological (e.g., biodiversity, nutrient cycling), physical (e.g., water quality, sediment stability), and climate-related (e.g., carbon sequestration) outcomes. We will also code the types of indicators used, methods of evaluation, and the timing of performance assessments. To identify knowledge trends and gaps, the evidence will be analyzed by geographic region, ecosystem type, spatial scale, and study design. This will allow us to identify clusters of evidence that may support future systematic reviews, and areas where empirical research is lacking. Visualizations such as heat maps, tables, and distribution summaries will be used to communicate these patterns.

The final evidence map will include a narrative summary and visual outputs highlighting the concentration and absence of evidence across key themes. It will also include reflections on policy and management implications. All metadata and study-level information will be made publicly available either through a data repository or as supplementary files in the published map.

## Electronic Supplementary Material

Below is the link to the electronic supplementary material.

Supplementary Material 1.

Supplementary Material 2.

Supplementary Material 3.

## Data Availability

All data generated or analysed during this study are included in this published article [and its supplementary information files].
