# Supplementary material for: What evidence exists for the environmental outcomes of nature-based aquaculture systems? a systematic map protocol
Source: Environ Evid. 2026 Jun 28;15:10. doi: 10.1186/s13750-026-00387-8 (PMC13576233; doi:10.1186/s13750-026-00387-8)
Supplement: Supplementary file 1 [file 13750_2026_387_MOESM2_ESM.pdf]

Additional File 1: ROSES for Systematic Map Protocols Compliance

ROSES ITEM	REQUIREMENT	WHERE REPORTED IN MANUSCRIPT	NOTES FOR COMPLIANCE
**TITLE**	Identify article as systematic map protocol	Page 1	Title clearly states systematic map protocol
**ABSTRACT BACKGROUND**	Context and rationale for the review	Page 1	Background explains the need to examine NbS in aquaculture
**ABSTRACT METHODS**	Search strategy, screening, extraction, synthesis	Page 1	Methods outline database searches, screening stages, inclusion criteria, and narrative synthesis
**INTRODUCTION RATIONALE**	Explain why review is needed	Pages 2–4	Clear justification of knowledge gaps in NbS aquaculture literature
**REVIEW QUESTION**	Explicit review question stated	Page 5	Research question clearly presented
**ELIGIBILITY CRITERIA**	Population, intervention, outcome, study types	Pages 6–7	Defined using aquaculture system, NbS intervention, and environmental outcomes
**LANGUAGE RESTRICTION**	State language inclusion and justification	Page 7	English language restriction stated
**SEARCH STRATEGY**	Describe search strings and strategy	Pages 8–9	Search strings and iterative refinement described
**DATABASES**	List publication databases searched	Page 8	Web of Science and Scopus included
**TIME PERIOD**	Define time coverage	Page 8	Search period defined from 2005 onwards

**GREY LITERATURE**	Additional search sources	Page 9	Google Scholar and specialist NbS platforms included
**SEARCH VALIDATION**	Benchmark articles used	Page 10	Ten benchmark studies used to test search completeness
**SCREENING PROCESS**	Title/abstract and full text screening stages	Pages 11–12	Two stage screening with pilot testing
**REVIEWER AGREEMENT**	Consistency test between reviewers	Page 11	Cohen’s Kappa used with threshold 75 percent
**CONFLICT RESOLUTION**	Handling reviewer disagreements	Page 11	Third reviewer decision process described
**REVIEWER CONFLICT OF INTEREST**	Authors not assessing their own studies	Page 12	Statement inserted to ensure unbiased inclusion decisions
**DATA EXTRACTION**	Describe extraction procedure	Page 12	Structured Google Form used
**DATA MANAGEMENT**	How extracted data are handled	Page 12	Data cross checked by MA
**EVIDENCE MAPPING**	Type of outputs produced	Page 13	Summary statistics and narrative synthesis
**EVIDENCE GAPS**	Identification of knowledge gaps	Page 13	Map designed to identify research gaps
**ADDITIONAL FILES**	Supplementary materials referenced	End section	Additional files provided for ROSES and search documentation

**DECLARATIONS**	Ethics, funding, competing interests	Final pages	Declarations included
**AUTHOR CONTRIBUTIONS**	Contributions to protocol manuscript	Final pages	Author roles stated
**REFERENCES**	[1] James, K.L., Randall, N.P. and Haddaway, N.R., 2016. A methodology for systematic mapping in environmental sciences. Environmental Evidence, 5(1), p.7.<br>[2] Bayliss, H.R., Haddaway, N.R., Eales, J., Frampton Environmental Evidence, 5(1), p.20.<br>[3] Haddaway, N.R., Kohl, C., da Silva, N.R., Schiema maps in environmental management. *Environmenta*<br>[4] Collaboration for Environmental Evidence. 2018. www.environmentalevidence.org/information-for-a<br>[5] Leeds Institute of Health Sciences. https://medhealth.leeds.ac.uk/info/639/information_specialists/1500/search_concept_tools. Accessed 12/11/2025.		
