# Supplementary material for: What evidence exists for the environmental outcomes of nature-based aquaculture systems? a systematic map protocol
Source: Environ Evid. 2026 Jun 28;15:10. doi: 10.1186/s13750-026-00387-8 (PMC13576233; doi:10.1186/s13750-026-00387-8)
Supplement: Supplementary file 2 [file 13750_2026_387_MOESM3_ESM.pdf]

Additional File 2: Evolution of search string.

What evidence exists for the environmental outcomes of nature-based aquaculture systems? A worldwide systematic map protocol

Additional File

This document summarizes the main steps taken to identify search terms and develop the search string through scoping exercises.

Scoping

An initial set of keywords was developed through expert consultation, a review of relevant academic literature, and early-stage discussions with stakeholder groups. These terms were aligned with the key elements of the research question, following the PIO structure:

- **Population**: Aquaculture systems located in marine, brackish, or freshwater environments
- **Intervention**: Implementation of nature-based or ecosystem-based approaches within aquaculture systems
- **Outcome**: Reported environmental outcomes resulting from the intervention

A scoping exercise was conducted in the **Web of Science Core Collection** to test the relevance, sensitivity, and specificity of various keyword combinations. Wildcards (e.g., "*") were used to capture singular and plural word variations. Alternative keywords were identified using subject-specific thesauri, controlled vocabularies, and manual review of relevant citations.

During scoping, a list of **ten benchmark articles**—representing key studies on nature-based aquaculture practices and their environmental impacts—was compiled. These articles were used to test the comprehensiveness of each iteration of the search string. Each test search was assessed based on its ability to retrieve the benchmark articles and recorded accordingly. The final search string was selected once all benchmark articles were successfully retrieved.

The finalized search string included terms linked to all three PIO elements. During testing, it was found that some aquaculture-related terms were too broad and resulted in a high volume of irrelevant records. To reduce noise while maintaining coverage, overly general terms were

removed or refined. Each term was tested individually; only those that added at least 50 relevant articles were retained.

Search specificity was further improved by applying structured Boolean logic in three ways:

1. Use of exact phrases with quotation marks (e.g., “aquaculture system”)
2. Use of the OR operator to include concept variations (e.g., “mangrove” OR “wetland”)
3. Use of the AND operator to combine core concepts (e.g., aquaculture AND biodiversity)

To ensure that relevant studies were not missed, the titles and abstracts of the first 100 results of each search iteration were reviewed. If less than 5 percent were deemed relevant, the search string was adjusted. Final refinement involved balancing search volume, sensitivity, and scope, ensuring comprehensive retrieval of relevant literature without excessive inclusion of off-topic studies.

The final search string was applied in the **Web of Science Core Collection** and **Scopus** databases and is documented in full in the main protocol.
